# PCR and Magnetic Bead-Mediated Target Capture for the Isolation of Short Interspersed Nucleotide Elements in Fishes

**DOI:** 10.3390/ijms13022048

**Published:** 2012-02-15

**Authors:** Dong Liu, Guoli Zhu, Wenqiao Tang, Jinquan Yang, Hongyi Guo

**Affiliations:** College of Fisheries and Life Science, Shanghai Ocean University, Shanghai, 201306, China; E-Mails: dliu@shou.edu.cn (D.L.); gl852@126.com (G.Z.); jqyang@shou.edu.cn (J.Y.); hy-guo@shou.edu.cn (H.G.)

**Keywords:** transposable element, SINE, tRNA, *Coilia nasus*

## Abstract

Short interspersed nucleotide elements (SINEs), a type of retrotransposon, are widely distributed in various genomes with multiple copies arranged in different orientations, and cause changes to genes and genomes during evolutionary history. This can provide the basis for determining genome diversity, genetic variation and molecular phylogeny, *etc*. SINE DNA is transcribed into RNA by polymerase III from an internal promoter, which is composed of two conserved boxes, box A and box B. Here we present an approach to isolate novel SINEs based on these promoter elements. Box A of a SINE is obtained via PCR with only one primer identical to box B (B-PCR). Box B and its downstream sequence are acquired by PCR with one primer corresponding to box A (A-PCR). The SINE clone produced by A-PCR is selected as a template to label a probe with biotin. The full-length SINEs are isolated from the genomic pool through complex capture using the biotinylated probe bound to magnetic particles. Using this approach, a novel SINE family, Cn-SINE, from the genomes of *Coilia nasus*, was isolated. The members are 180–360 bp long. Sequence homology suggests that Cn-SINEs evolved from a leucine tRNA gene. This is the first report of a tRNA^Leu^-related SINE obtained without the use of a genomic library or inverse PCR. These results provide new insights into the origin of SINEs.

## 1. Introduction

Short Interspersed Nucleotide Elements (SINEs) are a class of retrotransposons widely distributed in eukaryotic genomes. They integrate into a new genomic locus through RNA reverse transcription [1]. Most SINEs are 80–400 base-pair (bp) long, with more than 10^4^ copies per genome in which SINEs exist. They consist of three regions: the 5′-terminal head, the body, and the 3′-terminal tail [2]. The heads of all currently known SINEs derive from one of the three types of RNA: tRNA, 7SL RNA, or 5S rRNA. The body is specific to the particular family, is of unknown origin, and unrelated to tRNA. The tail includes a variable number of simple repeats [3]. 7SL RNA-derived SINEs are present only in primate and rodent genomes [4], 5S rRNA-related SINEs are known from a few fishes and bats [5], while SINEs derived from tRNA gene are widely disseminated among organisms [2,5]. Transcription of SINEs is done by polymerase III via the internal promoters of tRNA-related regions, and the reverse transcription is inserted into a new integration site by a “copy-and-paste” mechanism, resulting in the chromosomal changes which provide the remarkable diversity of eukaryotic genomes [6]. SINEs have influenced genomic evolution either through alterations of gene functions or as a source of new genetic material that allows for the emergence of genetic novelty [7]. The generation of allelic diversity through the insertion of SINEs provides useful molecular markers for studies of species identification, phylogeny, evolution, and population biology [8–10].

Although numerous copies of SINEs in certain eukaryotic genomes compose a SINE family, these copies are not identical and their sequences vary by 5–35% [2]. Different species rarely share the same SINE family, except when there has been horizontal transfer of SINEs among species [11]. As a result, it is impossible to isolate SINEs from various species using only one method. To date, several methods have been developed to detect SINEs in genomic DNA [12–16]. Early methods for the isolation of SINEs used polymerase III transcription of total genomic DNA *in vitro* to yield RNA complementary to DNA fragments containing SINEs; subsequently this was used as a template for a mRNA probe, and the labeled probe was used to screen genomic libraries, but the resulting probes are not very specific and their efficiency of binding target sequences is low. It is important to obtain a valid probe specific to a particular region of the repeated elements, therefore, Borodulina and Kramerov [12], taking advantage of the conserved boxes A and B of the tRNA-derived region, proposed a PCR-based approach (“AB-PCR”) to obtain the internal sequences between the two boxes, then these sequences were used as a template to label a hybridization probe for scanning genomic DNA. The internal sequence is short (~50 bp) and its efficiency in determining SINE in genome is not high. Therefore, they modified the “AB-PCR” method such that the products of a PCR with a primer complementary to box A, rather than genomic DNA, were used to construct a library. Since the PCR library is enriched in genomic regions containing SINEs, the efficiency of SINE isolation using a probe to scan the library is increased in the modified approach [13]. These methods seem to only isolate SINEs derived from tRNA^Tyr^, tRNA^Ala^, or tRNA^Arg^ genes [9,12,13,17]. Using AB-PCR, Han and He [18] additionally carried out inverse PCR to obtain the sequences flanking the spacer region between boxes A and B. The resulting sequences were used as a template for probe labeling, and SINEs in Cyprinid fish were identified via hybridization to a genomic library. Tong *et al*. [14] used a biotinylated probe bound to magnetic particles to capture target DNA fragments containing SINEs from an unknown genome after obtaining the sequence of the probe by AB-PCR together with inverse PCR as mentioned above.

The approaches described above mostly depend on a probe that is specific to a particular region of SINEs to isolate repeated elements. Here we describe a new, simple, and efficient method to obtain probe sequence for rapid isolation of SINEs, in which inverse PCR, library construction, and screening are entirely omitted. This method is based on two PCRs with only one primer for each to make DNA fragments containing SINEs as a template for a biotin-labeled probe. The probe is then bound to a magnetic particle and used to isolate SINEs from genomic DNA. Here we used the grenadier anchovy *Coilia nasus* as an example, isolating novel SINEs from its genome.

## 2. Results and Discussion

### 2.1. Results

#### 2.1.1. B-PCR

**Figure 1:**
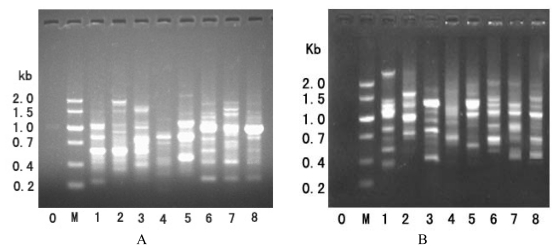
Results of B-PCR (**A**) and A-PCR (**B**) of genomic DNAs from various species. Well 0 is the control; M, size markers; 1, *Boleophthalmus pectinirostris*; 2, *Pelteobagrus fulvidraco*; 3, *Anchoviella Chinensis*; 4, *Thryssa kammalensis*; 5, *Setipinna taty*; 6, *Coilia grayi*; 7, *Coilia mystus*; and 8, *Coilia nasus*.

The results of B-PCR of genomic DNA from eight fishes are shown in Figure 1. This PCR was performed with only one primer, corresponding box B of the tRNA-Leu-like region. The number of resulting bands varied from three to six among the fish genomes (Figure 1A). *T. kammalensis* gave the fewest bands, where one was intense and two were weak. The shortest band was ~300 bp, and was present in *C. nasus*, *C. mystus*, *C. grayi*, and *B. pectinirostris*. The longest fragment was ~2000 bp, observed in *P. fulvidraco*. The amplified patterns in the B-PCR of these fishes may be due to genomic fragments that included at least two “tail-to-tail” oriented SINE copies inserted not far from each other.

**Figure 2:**
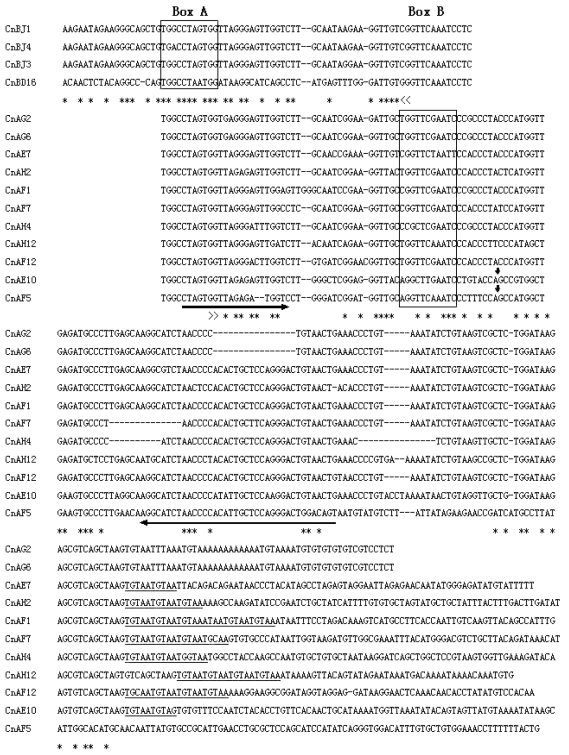
Results of two single-primer PCRs. Alignment of clone sequences from PCR products that were obtained using primer B (4 upper sequences) or primer A (11 lower sequences). The primer sequences are replaced by the following marks: >>, primer A and <<, primer B. Regions of box A and box B are enclosed by solid lines. Direct repeats are underlined. Asterisks indicate identical nucleotides; dash rules denote gaps; a pair of internal primers used for probe biotinylation is marked with lateral arrows; and the insertion site is shown by vertical arrows. GenBank accession numbers: JQ083287–JQ083297.

*C. nasus* was selected as a representative species for further study. All the bands produced from this species by PCR were separately excised from the gel and purified, then propagated in *E. coli* DH5α cells. Positive clones with inserted fragments of the expected sizes were selected randomly and sequenced. Six of 15 sequenced clones revealed that they contained a box A (Figure 2). Three of the cloned target DNAs were identical and were therefore not included into the alignment shown in the Figure 2.

#### 2.1.2. A-PCR

**Figure 3:**
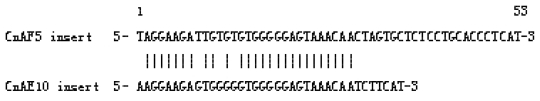
Alignment of the inserts in two clones obtained using primer A. Vertical lines indicate paired nucleotides.

Only the A primer to box A of tRNA-Leu-like region was used to perform PCR amplification of SINE copies from genomic DNA. The band patterns of PCR products are shown in Figure 1. Multiple bands were produced from one genome, suggesting that genomic DNA fragments contained SINE copies oriented in “head-to-head” arrangement, separated from each other by linker sequences of variable length. For further detailed study, the PCR bands obtained from the genome of *C. nasus* were excised from the gel, cloned, and sequenced. The sequence dataset revealed that these clones have characteristics of SINE elements (Figure 2), such as box B sited at the 5′ region, a tail region containing poly(A), A + T richness, or a 5-bp repeated motif (TGTAA)*_n_* in the 3′ region. This tandem motif may be produced by a tandem duplication event. The tRNA-related region was highly conserved between these clones obtained by A- and B-PCR. In the tRNA-unrelated region, however, these clones showed significant variety, especially in a large fragment inserted in the region of two clones (CnAE10 and CnAF5). Although the inserts are, respectively, 37 bp and 55 bp long, they share a common central domain (26/29 bp) (Figure 3). The conserved sequences indicate that the inserts originated from the same DNA sequence and subsequently evolved through mutation.

#### 2.1.3. Isolation of SINEs from the *C. nasus* Genome

**Figure 4:**
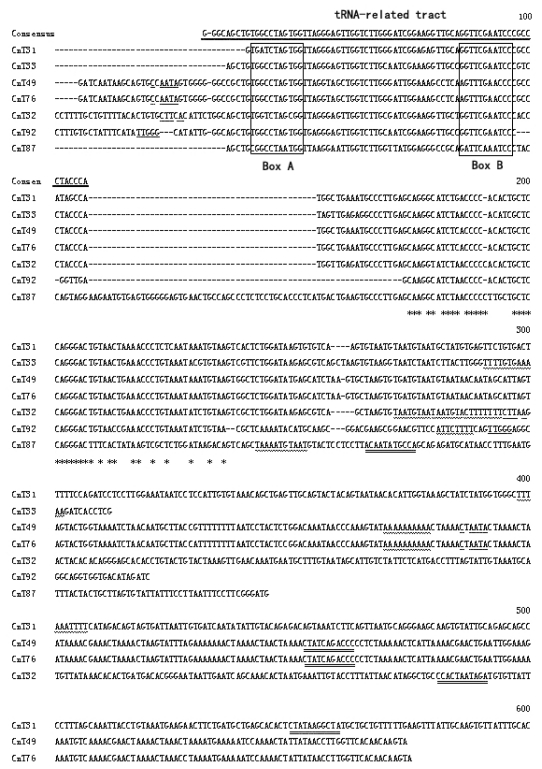
Cn-SINEs from *Coilia nasus*. The consensus sequences of the tRNA-related tract are shown on top and marked with thick bar. Boxes A and B are enclosed by solid lines. The target site duplications at each SINE locus are denoted by single underlines. A wavy line under nucleotides indicate poly(A) or AT-rich regions, and double lines under nucleotides show a reversed box A tract. Shaded nucleotides denote polymerase III transcription terminators. Asterisks indicate identical nucleotides; dashes denote gaps. GenBank accession numbers: JQ083280–JQ083286.

A total of seven clones that contained SINEs were isolated from *C. nasus* genomic DNA, using probe bound to magnetic particles. Alignment of these clones’ sequences showed that they have a 75-bp tRNA-related tract. In addition, a tRNA-unrelated region and a ~40-bp-long tail with an A/T-rich tract were also found in these clones, indicating that they are copies of a type of SINE (Figure 4). We used BLAST to search for sequences homologous to the SINEs from *C. nasus* in the NCBI databases [19]. No significant similarity was found except that a 386-bp fragment (position 415 to 800) of clone CnAE7 is homologous to long interspersed nuclear elements (LINEs) in the pufferfish, *Tetraodon nigroviridis* (position 913 to 524, GenBank accession: AJ312227) (data not shown). These clones thus belong to a new family of SINEs, which we name Cn-SINEs (SINEs from *C. nasus*). The members of this family are 180–360 bp long. We have deposited their sequences in the NCBI databases (GenBank accession: JQ083280–JQ083297).

**Figure 5:**
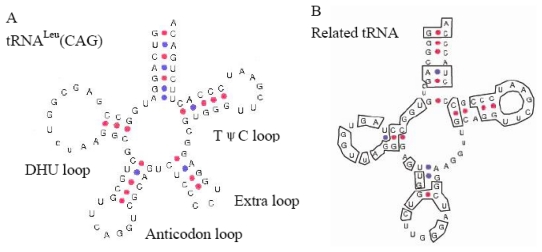
The secondary structure of tRNA^Leu^(CAG) in *Xenopus laevis* taken from [20] (**A**) and the tRNA^Leu^-related Cn-SINE family (**B**). The sequences identical to tRNA^Leu^(CAG) in Cn-SINE are boxed.

The BLAST homology search revealed that the tRNA-related region of the consensus Cn-SINE family was most similar to tRNA^Leu^ in *Xenopus laevis* (62%, not counting the extra arm-loop region) [20], and showed high sequence conservation (91%) in box A and box B relative to tRNA^Leu^ in *X. laevis* and *Drosophila melanogaster*. It is also 83% identical to the 5′ end of the *Hpa1*SINE isolated from *Parahucho perryi* [21]. The predicted secondary structure of the tRNA-derived region of Cn-SINE family has the common characteristics of tRNAs such as 5'pG, 3'CCA, and the arm and loop structures. These new structures lack an extra arm present in tRNA^Leu^ from *X. laevis*. However, the acceptor arm, 3-pair-bp-DHU arm, 5-pair-bp-TψC arm, and TψC loop of Cn-SINE are highly similar to that of the tRNA^Leu^ gene (Figure 5). This conservation of secondary structure suggests that the tRNA-related region of the Cn-SINE family likely evolved from the tRNA^Leu^ gene.

**Figure 6:**
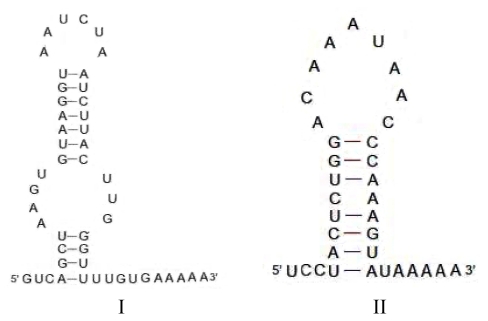
Putative secondary structure inferred from the tail region of Cn-SINEs. (**I**) with two loops inferred from the sequence of clone CnT33. (**II**) with one loop inferred from that of clone CnT49.

Analysis of the full-length SINEs isolated from *C. nasus* revealed direct repeats flanking each terminus of the SINEs in this family. A “TTT” sequence that acts as a strong polymerase III terminator in tRNA genes, including the tRNA^Leu^ gene [20], was found in the tail region, suggesting that transcription in SINE elements should terminate at this locus. The putative secondary structure of the tail region of Cn-SINE was as previously described [22]. Although two types (I and II) of loop structures were inferred from the sequences of the different clones, a hairpin structure was found in the tail with a string of A’s in the DNA template strand and the U’s in the RNA product (Figure 6). The rU-dA base pairs are exceptionally weak. This led polymerase III to pause at the rU-dA base pairs locus, allowed the RNA to dissociate from the DNA template strand and ensured the complete transcription of the SINEs. Interestingly, an imperfect inverse-repeated tract corresponding to box A was found in Cn-SINEs (Figure 4). It likely results from a head-to-head orientation of SINEs. This feature allowed us to isolate copies of these SINEs by use of a simple, single-primer PCR method.

### 2.2. Discussion

#### 2.2.1. Structure-Based PCR for Capturing New SINEs

In the present study, we exploited the structural features of SINEs to capture novel SINE families from the genome of *C. nasus*. In general, the known SINEs have the conserved boxes A and B, which serve as internal transcription promoters of polymerase III [12]. In addition some head-to-head/tail-to-tail-oriented members of SINE families are separated by short spacer sequences, although the majority of SINEs are widely scattered in the genome [13]. Members of the tRNA-derived SINE family may exist in distantly related fishes, arranged in head-to-head or tail-to-tail orientation. In our study, the presence of box A and box B sequences within the clones initially obtained via A/B-PCR does not prove that SINEs exist in the genome. Clones with the spacer sequence between box A and B of SINEs could be used as templates for biotinylated probes. This is an important step in using probes linked to magnetic particles to capture SINEs as described [14]. In the previous method, inverse PCR was used to obtain the sequence used as the template for making the biotin-labeled probe, with subsequent capture of genomic DNA fragments containing target sequence by the bead-linked probe. Our approach omitted inverse PCR, and only two PCRs were performed to obtain probe template for isolation of full-length SINEs, without genomic library construction or screening.

The Cn-SINE family that we isolated in this study has not been reported previously, and belongs to a novel family. This success in finding previously undescribed SINEs validates our approach. The box A and B sequences of Cn-SINEs are 91% identical to that of the tRNA^Leu^ genes of *Drosophila* and *Xenopus* [20,23], indicating that Cn-SINEs may have originated from tRNA^Leu^ genes. BLAST of the consensus tRNA-related sequence of Cn-SINEs against the NCBI databases shows 83% identity to the 5′-terminal section of *Hap*1 SINE (D49862, AB002416) obtained using *in vitro* synthesis of labeled RNA from *P. perryi* and *Oncorhynchus masou* DNAs [21], suggesting that SINEs derived from tRNA^Leu^ genes are likely to be present in various species. Successfully isolation of Cn-SINEs from *C. nasus* demonstrates that our method is a simple and rapid way to isolate tRNA^Leu^-related SINEs from the unsequenced genomes of these species. Borodulina and Kramerov [12], taking advantage of the conserved box A and box B, developed the “A-B” PCR approach to isolate SINEs from bat genomes, and other authors have used this method to isolate SINEs from various genomes [9,14,17]. We tried to isolate SINEs from *C. nasus* by “A-B” PCR, but failed to detect any (data not shown). To our knowledge, the B(A)-PCR method described in this paper is the first to discover a SINE family derived from a tRNA^Leu^ gene or a tRNA^Leu^-like gene.

#### 2.2.2. Origin of SINEs Isolated from *C. nasus*

More than 100 SINE families have been described from the genomes of various eukaryotes [24]. Of the previously known SINE families, most have a tRNA-derived tract in the 5′-end region of the repeated elements, and the tRNA genes of origin are commonly one of eight tRNA genes (Ala, Arg, Ile, Tyr, Gly, Ser, Lys, and Glu) [5]. For example, the CHRS family originated from tRNA^Glu^ in the genomes of artiodactyls and cetaceans [7]; the salmonid SINE families derived from a tRNA^Lys^ gene [25]; and a tRNA^Arg^-related region of the P.k.SINE family was described in bats [17]. The novel Cn-SINEs isolated from the *C. nasus* genome have a tRNA^Leu^-related region at their 5′ end, and their consensus tRNA-related sequence contains the conserved 11-bp box A and 11-bp box B separated by a 33-bp spacer similar to other SINE families [8]. The tRNA-unrelated region of Cn-SINEs varies in length mainly due to nucleotide indels, but high sequence identity (~90%) was found among these members of the Cn-SINE family, excluding the large inserted fragment (Figure 4; identical nucleotides are denoted by asterisks). A high degree of similarity was observed between the insertions of clones CnAE10 and CnF5, though they differed in length (Figure 3). These results suggest that the tRNA-unrelated regions of Cn-SINEs may be of common origin from DNA fragments, subsequently subjected to numerous mutational events during SINE evolution. There are other cases of a common central domain in the tRNA-unrelated region existing in various SINE families [26].

The Cn-SINEs have a 3′-terminal tail with poly(A), or an AT-rich region. The length of the tail region is 17–48 bp. Two types of secondary structures were inferred from the sequence of the tail region of SINEs in different clones (Figure 6), and both have a hairpin structure. This is required for efficient release of the transcript of DNA with SINE fragments. The hairpin structure is followed by a string of A’s in the tail. As the DNA template of SINE is transcribed, the poly(A) would let the RNA dissociate from the template and terminate transcription of SINE through a string of A-U base pairs formed between the A’s in the SINE template strand and the U’s in the transcription. Thus, transcription of the full SINE would probably be confined to special DNA fragments. The poly-A tail of a transcribed SINE will attach to the free “TTTT” site of the target DNA fragment, and act as a primer for reverse transcriptase to synthesize a new SINE insert in the target site [3]. Because the two strands of the “new” DNA are cut at staggered sites, the inserted SINE is flanked by small gaps which, when filled in by a host enzyme, lead to short target-site duplications (TSDs) at the inserted sites [27]. The Cn-SINEs have a perfect or irregular TSD flanking each SINE locus (Figure 4), revealing that in Cn-SINEs, amplification and integration, which are dependent on enzymes derived from the host genome and LINEs, occurred during evolution. SINEs and LINEs have similar 3′ tails, and SINEs acquire retropositional activity from reverse transcriptase generated by LINEs [14,17,28]. In our present study, one clone, CnAE7, has a 3′ terminal tail similar to LINEs in the pufferfish *Tetraodon nigroviridis*, implying that the 3′ tail of Cn-SINE family members was derived from LINEs. These and further studies may reveal the influence of these novel Cn-SINEs on the evolution of fish genomes as well as help explain how this type of retrotransposition originates from tRNA^Leu^ genes.

## 3. Experimental Section

### 3.1. Fish Samples and DNA Extraction

The organisms studied are eight species belonging to three families and three orders, representing various ecotypes of fish including freshwater, estuary, seawater, and anadromous populations. One individual of each of the eight species—*C. nasus*, *C. mystus*, *C. grayi*, *Setipinna taty*, *Thryssa kammalensis*, *Anchoviella chinensis*, *Pelteobagrus fulvidraco*, and *Boleophthalmus pectinirostris—*was first analyzed by PCR to detect the distribution of SINEs in the genome, and *C. nasus* (anadromous) was chosen as a representative species for further study. Species identification was carried out according to the literature [29–31], and samples were deposited in alcohol at the Laboratory of Ichthyology, Shanghai Ocean University, in China.

Genomic DNA was extracted from small amounts of ethanol-preserved muscle by proteinase K digestion in lysis buffer at 55 °C for 2–3 h, following the manufacturer’s protocols for the UNIQ-10 DNA Extraction Kit (Sangon, Shanghai, China). The purity of DNA preparations was determined using a BioPhotometer (Eppendorf, Hilden, Germany) and electrophoretic run on 1% agarose then stained in ethidium bromide. DNA was stored at −20 °C.

### 3.2. Design of B(A)-PCR, Cloning and Sequencing

**Figure 7:**
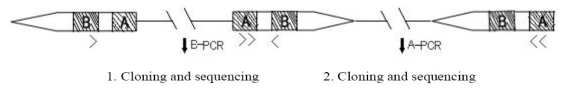
Scheme of primer design for short interspersed nucleotide elements (SINE) isolation using B-PCR and A-PCR. “<” and “>” denote the orientation in B PCR amplification; “<<” and “>>” denote the orientation in A PCR amplification. Shaded tracts show the boxes A and B in SINE. Number 1 and 2 indicate cloning and sequencing after PCR amplification.

We call this method B(A) PCR. The single primer was designed against the conserved box B or box A of the tRNA^Leu^ genes from various organisms. The SINE isolation strategy is shown in Figure 7. Two primers, complementary to box A and box B, were designed and used separately for single-primer PCR amplification. The B primer sequence was 5′-GAGGAYTTG AACC-3′, and the A primer sequence was 5′-TGGCCTAGTGG-3′. PCR reactions of 50 μL volume contained ~100 ng of DNA, a single primer at 0.5 μM, each dNTP at 0.4 μM, and 2 U *Taq*-plus polymerase (Tiangen, Beijing, China). PCR was carried out in an Eppendorf Mastercycler PCR System (Eppendorf, Hilden, Germany) as follows: 94 °C for 2 min, and 30 cycles of 94 °C for 30 s, 44 °C (B-PCR, and 43 °C for A-PCR) for 45 s, and 72 °C for 1 min. A final extension was performed at 72 °C for 10 min.

PCR products were separated by 1.0% agarose gel electrophoresis and visualized by ethidium bromide staining, then purified using a UNIQ-10 column PAGE gel DNA purification kit (Sangen, Shanghai, China) according to the manufacturer’s protocol. Purified products were inserted directly into the pMD19-T vector (Takara, Dalian, China) and then used to transform *E. coli* DH5α cells. The recombined clones were identified by blue/white screening, and picked randomly for sequencing. All clones were sequenced using vector-specific primers for M13 and SP6 sequences, and an automated DNA sequencer (ABI PRISM 3730).

### 3.3. Genomic DNA Enrichment

#### 3.3.1. Restriction Digestion of Genomic DNA

Genomic DNA (~1 μg) was completely digested for 2 h at 37 °C with *Bsp*1431 (Takara, Dalian, China) in a total volume of 100 μL. The fragments were separated by 1% agarose gel electrophoresis. The fragments between ~400 bp and ~2000 bp were purified from the gel using a gel DNA purification kit and suspended in 50 μL of distilled water.

#### 3.3.2. Ligation of DNA Fragments to the Adaptor

The adaptor is a partially double-stranded DNA composed of a long strand annealed to a short strand to yield *Bsp*1431-compatible cohesive ends (a 3′ 4-bp overhang). The adaptor sequence was as follows: 5′-GTAATACGACTCACTATAGGGCCGAGGT-3′, 3′-CCCGGCTCCACTAG-5′.

Excess double-stranded adaptor was added to the above 50 μL of prepared *Bsp*1431-digested DNA fragments in a 100 μL reaction mixture containing buffer and 20 units T4 DNA ligase (Takara, Dalian, China). The reaction proceeded overnight at 16 °C, and was then purified on a UNIQ-10 column, and finally dissolved in 50 μL of distilled water.

#### 3.3.3. PCR Amplification of Genomic DNA

The adaptor primer used for PCR amplification of genomic DNA corresponds to the 5′-end sequence of the long strand of the adaptor. The primer sequence was 5′-GTAATACGACTCACTATA GGGC-3′. The adaptor-ligated DNAs were amplified by PCR in a 20 μL volume composed of 15.6 μL distilled water, 2.5 μL 10× PCR Buffer, 0.5 μL of 10 μM primer, 0.2 μL of 10 mM dNTPs, 0.2 μL of *Taq* DNA polymerase, and 1 μL of the adaptor ligation product. The PCR began at 72 °C for 5 min to extend the 3′ end of the adaptor fraction, followed by 17 cycles of 94 °C for 30 s, 58 °C for 45 s, 72 °C for 90 s; final extension was 72 °C for 10 min. Five separate PCR products were pooled to 100 μL and purified using a column purification kit and finally suspended in 100 μL of distilled water.

### 3.4. Magnetic Particle Isolation of SINEs

The magnetic particle system was used according to the manufacturer’s instructions (MagneSphere magnetic separation products; Promega, Germany). The isolation strategy for SINEs is described elsewhere [14].

#### 3.4.1. Probe

The forward (F) and reverse (R) PCR primers used to synthesize biotin-labeled probes were designed by Primer premier 5.0 [32] based on an internal region of a SINE from the clone CnAG2 obtained by B-PCR (Figure 2). The F primer sequence was 5′-AGTGGTGAGGGAGTTGGTCTT-3′. The R primer sequence was 5′-GGGTTTCAGTTACAGGGGTTAG-3′. Biotin was added to the 5′ end of the F primer. A biotinylated probe was obtained via PCR with the biotin-labeled F and R primers and clone CnAG2 as the template. PCR was performed and products were purified as described above, except that the annealing temperature was 56 °C.

#### 3.4.2. Isolation of Target Sequences

The denatured biotinylated probe above was added to the tube containing the denatured genomic fragments with adaptor at their ends. These were annealed at 55 °C for 2 h then incubated at room temperature until completely cooled to allow the biotin-labeled probe to hybridize with the adaptor DNA fragments.

A volume of 0.6 mL of MagneSphere magnetic particles was washed three times with 0.5× SSC (300 μL per wash), and the wash solution was carefully removed using the magnetic stand. The washed magnetic particles were resuspended in 100 μL of 0.5× SSC, added to the prepared probe-adaptor DNA hybrid solution, and incubated at room temperature for 30 min so that the biotin present in 5′ end of the adaptor DNA-hybrid probe specifically attached to the magnetic particles.

The supernatant containing DNA strands not attached to the magnetic beads was removed by washing four times with 0.1× SSC (300 μL per wash). The magnetic stand was used to separate the magnetic beads from the supernatant. Finally, the probe-target DNA complexes were eluted from the magnetic particles in 50 μL of distilled water at 94 °C for 5 min.

#### 3.4.3. Adaptor PCR, Cloning and Sequencing

The target DNA fragments were used as a template for PCR with the adaptor primer. The PCR volume and profile were as described above. The PCR products were inserted into *E. coli* DH5α cells for propagation. The insert sizes in many single bacterial clones were directly determined by PCR with the S7 and R47 pMD-19 T vector primers. The clones having inserts longer than 300 bp were chosen randomly and sequenced.

## 4. Conclusions

Using a developed protocol that combines PCR and magnetic bead, we isolated and identified a novel Cn-SINE family from the genome of *C. nasus* without the use of a genomic library or inverse PCR. These members of this family derived from tRNA, and consisted of a 5′-terminal head, a body, and a 3′-terminal tail. Sequence homology of the 5′-terminal head of the family was similar to a tRNA-leucine-like region, indicating that this family evolved from the tRNA-Leu gene. High diversity was observed in the regions of the body and the 3′-terminal tail, making the Cn-SINEs molecular markers with the future study about population genetic structures of the genome of *C. nasus*. All of this knowledge will increase our understanding of the origin of SINEs and its biological roles of fish evolutionary history.
